# Dynamic Performance Assessment of Hospitals by Applying Credibility-Based Fuzzy Window Data Envelopment Analysis

**DOI:** 10.3390/healthcare10050876

**Published:** 2022-05-09

**Authors:** Pejman Peykani, Elaheh Memar-Masjed, Nasim Arabjazi, Mirpouya Mirmozaffari

**Affiliations:** 1 School of Industrial Engineering, Iran University of Science and Technology, Tehran 1684613114, Iran; pejman.peykani@yahoo.com; 2 Department of Industrial Engineering, Faculty of Engineering, Ferdowsi University of Mashhad, Mashhad 9177948974, Iran; e.memarmasjed@gmail.com; 3 Department of Mathematics, Faculty of Science, Science and Research Branch, Islamic Azad University, Tehran 1477893855, Iran; nasim.arabjazi@srbiau.ac.ir; 4 Department of Industrial Engineering, Dalhousie University, 5269 Morris Street, Halifax, NS B3H 4R2, Canada

**Keywords:** hospital performance assessment, data envelopment analysis, window analysis, fuzzy optimization, linguistic variables, credibility theory

## Abstract

The goal of the current research is to propose the credibility-based fuzzy window data envelopment analysis (CFWDEA) approach as a novel method for the dynamic performance evaluation of hospitals during different periods under data ambiguity and linguistic variables. To reach this goal, a data envelopment analysis (DEA) method, a window analysis technique, a possibilistic programming approach, credibility theory, and chance-constrained programming (CCP) are employed. In addition, the applicability and efficacy of the proposed CFWDEA approach are illustrated utilizing a real data set to evaluate the performance of hospitals in the USA. It should be explained that three inputs including the number of beds, labor-related expenses, patient care supplies, and other expenses as well as three outputs including the number of outpatient department visits, the number of inpatient department admissions, and overall patient satisfaction level, are considered for the dynamic performance appraisal of hospitals. The experimental results show the usefulness of the CFWDEA method for the evaluation and ranking of hospitals in the presence of fuzzy data, linguistic variables, and epistemic uncertainty.

## 1. Introduction

The hospital, as one of the most important and main parts of the health care system, has a prominent and significant role in the performance of health care networks [1,2,3,4,5]. As observed during the coronavirus pandemic, the quality level of hospital performance had a remarkable effect on patient mortality rate. Thus, proposing an effective method to assess the performance and productivity of hospitals is one of the most important issues in health care literature [6,7,8,9,10,11,12,13]. Data envelopment analysis (DEA) is one of the popular and applicable non-parametric mathematical programming methods that are widely employed by many researchers in the health care field to appraise the productivity and performance of hospitals and their departments [14,15,16,17,18,19,20,21,22,23,24]. DEA is one of the most powerful and effective multi criteria decision making (MCDM) approaches for performance assessment, benchmarking, and ranking the peer decision-making units (DMUs) in the presence of multiple inputs and outputs. Furthermore, DEA is capable of identifying the efficient frontier (EF) of a production possibility set (PPS). The EF represents the maximal output attainable from each input level [25,26,27,28,29].

**Figure 1:**
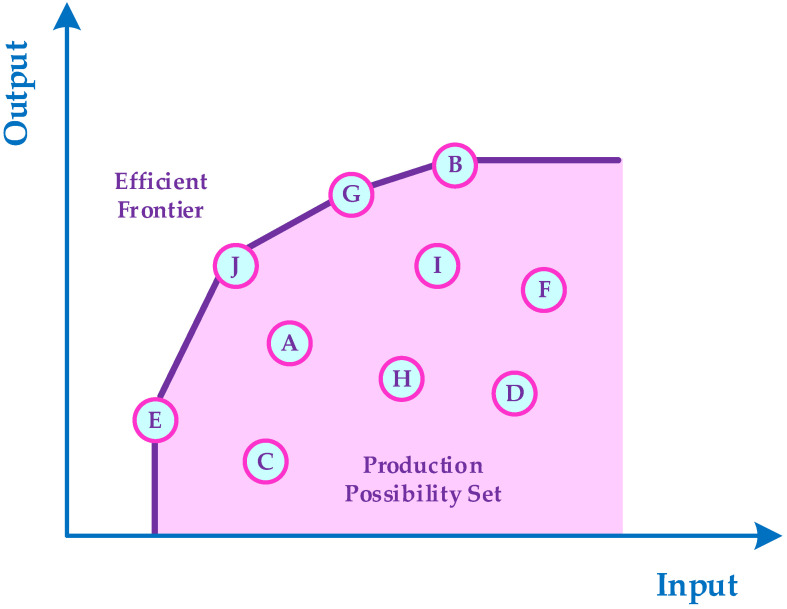
The graphical presentation of DEA approach for performance evaluation of DMUs.

Figure 1 illustrates the EF and PPS, where one input and one output are considered. Based on the DEA approach, the DMUs E, J, G, and B are technically efficient whereas the DMUs C, A, H, D, I, and F are technically inefficient in Figure 1.

Notably, one of the main problems and issues in the performance assessment of hospitals in real-life case studies is to identify the trend and the effect of time variations as well as the dynamic changes in the performance level of each hospital over time periods. In addition, some of the variables, such as overall patient satisfaction level as an important criterion for hospital performance appraisal, are linguistic variables that can be converted to fuzzy variables. Since the conventional and traditional DEA models are not capable of being applied under a panel data and fuzzy environment, proposing, and applying new data envelopment analysis models that can measure the dynamic performance of hospitals under data ambiguity during different periods seems to be essential.

Accordingly, in this research, the credibility-based fuzzy window data envelopment analysis (CFWDEA) approach is presented for the dynamic performance appraisal of hospitals over time under linguistic variables and data ambiguity. It should be explained that to propose the CFWDEA method, data envelopment analysis, window analysis, possibilistic programming, credibility theory, and chance-constrained programming (CCP) are applied. Moreover, the proposed CFWDEA approach is implemented in a real-life case study for assessing the dynamic performance of six hospitals in the USA during six different periods.

The rest of this paper is organized as follows. The applications of the window data envelopment analysis (WDEA) method in the health care field, as well as literature gaps, are presented in Section 2. Then, the credibility-based fuzzy window DEA approach for the dynamic performance appraisal of hospitals in the presence of linguistic variables and fuzzy panel data are proposed in Section 3. Furthermore, the proposed CFWDEA approach is applied to a real-world case study and the experimental results are analyzed in Section 4. Finally, conclusions as well as some suggestions and directions for future research are introduced in Section 5.

## 2. Literature Review

In this section, the literature review of window data envelopment analysis applications in health care systems is presented. Moreover, the literature research gaps, which this study addresses, are introduced. Accordingly, the characteristics of window DEA studies in health care area including the basic DEA model, the case study, the application location, and the data type are presented in Table 1.

As summarized in Table 1, all the existing window DEA studies are implemented in health care systems and omit the uncertainty of data. As a result, presenting an effective and novel approach that is capable of being applied for the dynamic performance assessment of hospitals during different periods under data ambiguity and linguistic variables is needed. Thus, as is seen in the last row of Table 1, in this research, the credibility-based fuzzy window DEA approach is proposed to evaluate the dynamic performance of hospitals in the presence of fuzzy panel data.

## 3. The Proposed Approach

**Figure 2:**
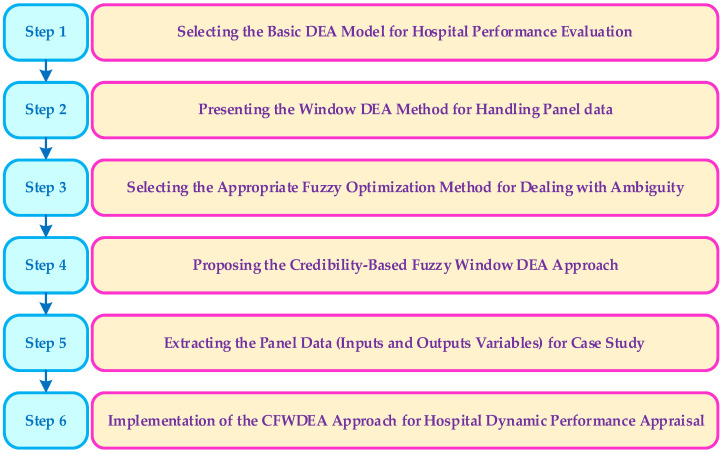
The schematic summary of all steps in the proposed CFWDEA approach.

In this section, the credibility-based fuzzy window data envelopment analysis approach is proposed step by step. It should be explained that at the first step, the classic DEA model under constant returns to scale (CRS) assumption is introduced. Then, using window analysis method, the traditional DEA model is developed under panel data. In the following, the window DEA model is prepared for considering ambiguity in all inputs and outputs. Finally, possibilistic programming, credibility theory, and chance-constrained programming are utilized to present the CFWDEA approach that is capable of being used in the presence of fuzzy panel data. The methodology of the paper is illustrated in Figure 2.

**Figure 3:**
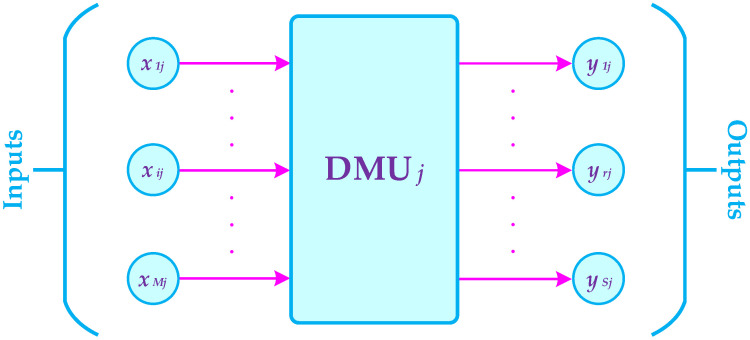
The presentation of homogeneous decision-making units in DEA method.

Now, according to Figure 3, suppose that there are N homogeneous decision-making units $DMU_{j}(j=1,2,\ldots ,N)$ that convert M inputs $x_{ij}(i=1,2,\ldots ,M)$ into S outputs $y_{rj}(r=1,2,\ldots ,S)$. In addition, the non-negative weights $P_{i}$ and $Q_{r}$ are assigned to inputs and outputs, respectively.

The efficiency score of specific $DMU_{d}$ that is an under evaluation DMU, can be measured by applying the following linear problem. It should be noted that Model (1) is called the multiplier form of input oriented CCR model [25].

(1) $$Max\;\sum_{r=1}^{S}\;y_{rd}Q_{r}$$

$$S.t.\;\sum_{r=1}^{S}\;y_{rj}\;Q_{r}-\sum_{i=1}^{M}\;x_{ij}\;P_{i}\leq 0,\;\forall j$$

$$\sum_{i=1}^{M}\;x_{id}\;P_{i}=1$$

$$P_{i},\;Q_{r}\geq 0,\;\forall i,\;r$$

Notably, by combining window analysis method and DEA model, the window DEA approach can be obtained that is capable to be used for dynamic performance evaluation of DMUs under panel data and different periods [48,49,50,51,52]. To present the WDEA model, suppose that all homogenous decision-making units $DMU_{j}(j=1,2,\ldots ,N)$ are observed in δ(t=1,2,…,δ) periods. Furthermore, let $k_{z}$ denote the window start in period k(1≤k≤δ) with width z(1≤z≤δ−k). It should be explained that the number of windows (α), the number of different DMUs per window (β), and the total number of different DMUs (λ) are calculated by α=δ−z+1, β=zN, and λ=αβ, respectively [53]. Accordingly, the window DEA approach for dynamic performance measurement of $DMU_{dk_{z}}$ is introduced as Model (2).

(2) $$Max\;\sum_{r=1}^{S}\;y_{rdk_{z}}Q_{r}$$

$$S.t.\;\sum_{r=1}^{S}\;y_{rjk_{z}}\;Q_{r}-\sum_{i=1}^{M}\;x_{ijk_{z}}\;P_{i}\leq 0,\;\forall j$$

$$\sum_{i=1}^{M}\;x_{idk_{z}}\;P_{i}=1$$

$$P_{i},\;Q_{r}\geq 0,\;\forall i,\;r$$

**Figure 4:**
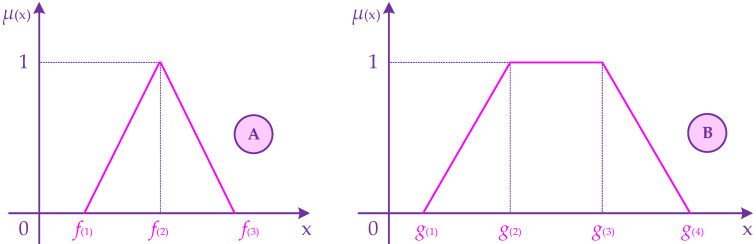
The representation of triangular (**A**) and trapezoidal (**B**) fuzzy numbers.

Now, assume that the inputs and outputs of window DEA approach are tainted by uncertainty. It is noteworthy that triangular fuzzy number (TRFN) and trapezoidal fuzzy number (TLFN) are the most popular and applicable fuzzy number in fuzzy mathematical field. Figure 4 presents the membership function curve of TRFN $\tilde{f}(f^{\left(1\right)},f^{\left(2\right)},f^{\left(3\right)})$, $f^{\left(1\right)}\leq f^{\left(2\right)}\leq f^{\left(3\right)}$ and TLFN $\tilde{g}(g^{\left(1\right)},g^{\left(2\right)},g^{\left(3\right)},g^{\left(4\right)})$, $g^{\left(1\right)}\leq g^{\left(2\right)}\leq g^{\left(3\right)}\leq g^{\left(4\right)}$.

To deal with the uncertainty of inputs and outputs, the objective function is converted into constraint. In addition, an equal constraint become a less than or equal constraint [54,55,56,57]. By assuming the fuzzy inputs and fuzzy outputs have a trapezoidal distribution ${\tilde{x}}_{ij}(x_{ij}^{\left(1\right)},x_{ij}^{\left(2\right)},x_{ij}^{\left(3\right)},x_{ij}^{\left(4\right)})$ and ${\tilde{y}}_{rj}(y_{rj}^{\left(1\right)},y_{rj}^{\left(2\right)},y_{rj}^{\left(3\right)},y_{rj}^{\left(4\right)})$ in which $x_{ij}^{\left(1\right)}\leq x_{ij}^{\left(2\right)}\leq x_{ij}^{\left(3\right)}\leq x_{ij}^{\left(4\right)}$ and $y_{rj}^{\left(1\right)}\leq y_{rj}^{\left(2\right)}\leq y_{rj}^{\left(3\right)}\leq y_{rj}^{\left(4\right)}$, the uncertain window data envelopment analysis (UWDEA) model under fuzzy panel data can be considered as Model (3).

(3) Max G

$$S.t.\;\sum_{r=1}^{S}\;{\tilde{y}}_{rdk_{z}}Q_{r}\geq G$$

$$\sum_{r=1}^{S}\;{\tilde{y}}_{rjk_{z}}\;Q_{r}-\sum_{i=1}^{M}\;{\tilde{x}}_{ijk_{z}}\;P_{i}\leq 0,\;\forall j$$

$$\sum_{i=1}^{M}\;{\tilde{x}}_{idk_{z}}\;P_{i}\leq 1$$

$$P_{i},\;Q_{r}\geq 0,\;\forall i,\;r$$

In order to deal with data uncertainty in constraints, credibility-based fuzzy chance-constrained programming (CFCCP) approach is used [58,59,60,61,62,63,64,65]. Let $\tilde{\omega }$ be a trapezoidal fuzzy variable on the possibility space (Φ,P(Φ),Pos) and ϕ be a crisp number. According to the CFCCP approach, the credibility (*Cr*) of fuzzy events $\left\{\tilde{\omega }\leq \phi \right\}$ and $\left\{\tilde{\omega }\geq \phi \right\}$ at the desired confidence level (ξ) are presented in Equations (4) and (5), respectively.

(4) $$Cr\{\tilde{\omega }\leq \phi \}\geq \xi \;\Leftrightarrow \left\{ \begin{array}{ll} (1-2\xi )\omega^{\left(1\right)}+2\xi \omega^{\left(2\right)}\leq \phi \; & if\;\xi \leq 0.5;\\ (2-2\xi )\omega^{\left(3\right)}+(2\xi -1)\omega^{\left(4\right)}\leq \phi \; & if\;\xi >0.5. \end{array}\right.$$

(5) $$Cr\{\tilde{\omega }\geq \phi \}\geq \xi \;\Leftrightarrow \left\{ \begin{array}{ll} 2\xi \omega^{\left(3\right)}+(1-2\xi )\omega^{\left(4\right)}\geq \phi \; & if\;\xi \leq 0.5;\\ (2\xi -1)\omega^{\left(1\right)}+(2-2\xi )\omega^{\left(2\right)}\geq \phi \; & if\;\xi >0.5. \end{array}\right.$$

As it can be seen in Equations (4) and (5), for the confidence levels of greater or less than 0.5, an equivalent crisp of fuzzy chance constraints (FCC) would be different. Now, by applying CFCCP approach, the credibility-based fuzzy window DEA model for ξ≤0.5 and ξ>0.5 are defined as Models (6) and Model (7), respectively.

(6) $$Max\;\underline{G}$$

$$S.t.\;\sum_{r=1}^{S}\left(\left(2\xi \right)y_{rdk_{z}}^{\left(3\right)}+\left(1-2\xi \right)y_{rdk_{z}}^{\left(4\right)}\right)Q_{r}\geq \underline{G}$$

$$\sum_{r=1}^{S}\left(\left(1-2\xi \right)y_{rjk_{z}}^{\left(1\right)}+\left(2\xi \right)y_{rjk_{z}}^{\left(2\right)}\right)\;Q_{r}-\sum_{i=1}^{M}\left(\left(2\xi \right)x_{ijk_{z}}^{\left(3\right)}+\left(1-2\xi \right)x_{ijk_{z}}^{\left(4\right)}\right)\;P_{i}\leq 0,\;\forall j$$

$$\sum_{i=1}^{M}\left(\left(1-2\xi \right)x_{idk_{z}}^{\left(1\right)}+\left(2\xi \right)x_{idk_{z}}^{\left(2\right)}\right)\;P_{i}\leq 1$$

$$P_{i},\;Q_{r}\geq 0,\;\forall i,\;r$$

(7) $$Max\;\bar{G}$$

$$S.t.\;\sum_{r=1}^{S}\left(\left(2\xi -1\right)y_{rdk_{z}}^{\left(1\right)}+\left(2-2\xi \right)y_{rdk_{z}}^{\left(2\right)}\right)Q_{r}\geq \bar{G}$$

$$\sum_{r=1}^{S}\left(\left(2-2\xi \right)y_{rjk_{z}}^{\left(3\right)}+\left(2\xi -1\right)y_{rjk_{z}}^{\left(4\right)}\right)\;Q_{r}-\sum_{i=1}^{M}\left(\left(2\xi -1\right)x_{ijk_{z}}^{\left(1\right)}+\left(2-2\xi \right)x_{ijk_{z}}^{\left(2\right)}\right)\;P_{i}\leq 0,\;\forall j$$

$$\sum_{i=1}^{M}\left(\left(2-2\xi \right)x_{idk_{z}}^{\left(3\right)}+\left(2\xi -1\right)x_{idk_{z}}^{\left(4\right)}\right)\;P_{i}\leq 1$$

$$P_{i},\;Q_{r}\geq 0,\;\forall i,\;r$$

Notably, since TRFN is a special case of TLFN, the proposed credibility-based fuzzy window DEA approach can be easily used in the presence of triangular fuzzy data.

## 4. Case Study and Experimental Results

**Figure 5:**
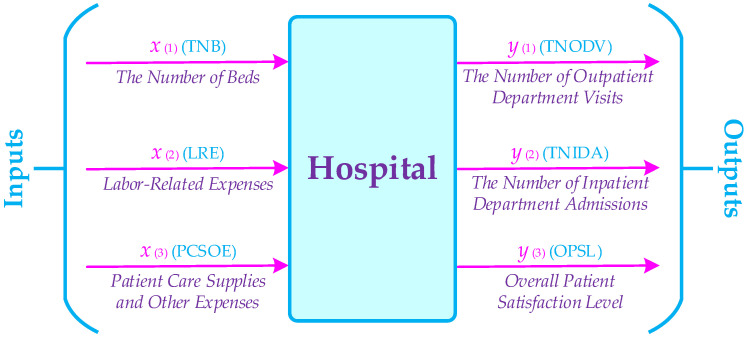
The inputs and outputs of CFWDEA model for health care case study.

In this section, the implementation of the proposed CFWDEA approach for a real-word case study is introduced. Accordingly, a real data set related to six hospitals from the USA for six different periods (2010–2015) is extracted. The inputs and outputs of the CFWDEA approach for hospital dynamic performance evaluation are presented in Figure 5 and Table 2.

It should be explained that all input and output data except the overall patient satisfaction are crisp values. The overall patient satisfaction level is reported with linguistic variables and their equivalent fuzzy numbers are introduced in Table 3 [66]. Finally, by setting the width of the window to three periods, the results of the credibility-based fuzzy window DEA approach for different confidence levels, including 0%, 20%, 40%, 60%, 80%, and 100% are reported in Table 4, Table 5, Table 6, Table 7, Table 8 and Table 9, respectively.

**Figure 6:**
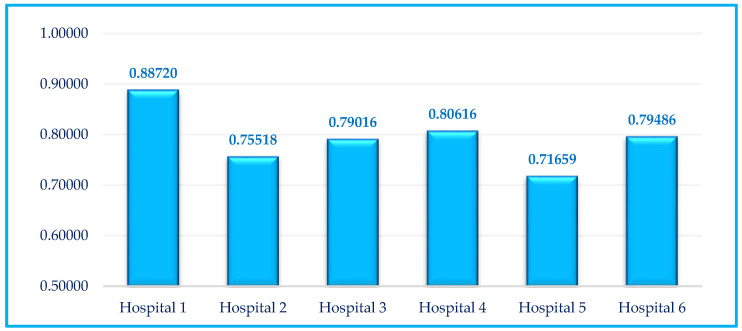
The total average CFWDEA results of hospitals.

Notably, since the width of the window is set to three periods, the number of windows, the number of different hospitals per window, and the total number of different hospitals are calculated as α=6−3+1=4, β=3×6=18, and λ=4×18=72, respectively. As is seen in Table 4, Table 5, Table 6, Table 7, Table 8 and Table 9, by increasing the confidence level from 0% to 100%, the results of the credibility-based fuzzy window DEA approach are decreased. Note that in addition to measuring the performance score of each hospital per window, three types of average scores, including the average performance scores of hospitals for all periods, the average performance scores of hospitals for all windows, and the average of all performance scores for each hospital are calculated. The total average results of all hospitals based on the CFWDEA approach are reported in Figure 6.

As can be seen in Figure 6, the full ranking of hospitals is obtained as Hospital 1, Hospital 4, Hospital 6, Hospital 3, Hospital 2, and Hospital 5, respectively. It is noteworthy that the highest efficiency score for all hospitals in all periods is obtained for Hospital 1 in Period 2. An examination of the data shows that the minimum amount of labor-related expenses (×2) as well as patient care supplies and other expenses (×3) for all hospitals in all periods is related to Hospital 1 in Period 2, which is equal to 3,778,001 and 2,036,342, respectively. Since Hospital 1 has the best overall performance in comparison with the other hospitals over a time horizon, the performance and planning of this hospital can be analyzed to be the benchmark for other hospital managements.

## 5. Conclusions and Future Research Directions

So far, various types of data including crisp data versus uncertain data (stochastic, fuzzy, interval, and mixed), cross-sectional data versus panel data, and quantitative data versus linguistic data have been used in the performance evaluation of hospitals. In this study, using a DEA model, a window analysis method, and credibility-based fuzzy chance-constrained programming, a novel and effective method is presented to evaluate the dynamic performance of hospitals in the presence of fuzzy panel data. Since utilizing linguistic variables allows the patients to easily represent their opinion about the provided services, the overall patient satisfaction is recorded with linguistic variables. The main advantages of the proposed CWFDEA approach can be mentioned as follows: the linearity of the mathematical models, the capability to fully rank all hospitals under data ambiguity, and the ability to examine the dynamic changes of the performance of each hospital over a time horizon. Moreover, implementation of the CWFDEA approach can increase the discrimination power by increasing the number of hospitals when a limited number of hospitals is available. For the future research, a robust optimization approach [67,68,69,70,71,72,73], uncertain theory [74,75,76,77,78], and Z-number theory [79,80,81,82,83,84,85] can be utilized in order to deal with data uncertainty.

## Figures and Tables

**Table 1:** The application of window DEA approach in health care systems: a literature review.

Year	Research	DEA Model	Case Study (Location)	Data Type
2004	Gannon [30]	CCR *	Hospital (Ireland)	Crisp
2005	Ozcan et al. [31]	BCC *	Mental Health Service (USA)	Crisp
2009	Kazley and Ozcan [32]	CCR	Hospital (USA)	Crisp
2009	Weng et al. [33]	CCR	Hospital (USA)	Crisp
2017	Flokou et al. [34]	BCC	Public Hospital Sector (Greece)	Crisp
2017	Jia and Yuan [35]	BCC	Multi-Branched Hospital (China)	Crisp
2017	Klangrahad [36]	BCC	Hospital (Thailand)	Crisp
2017	Mirmozaffari and Alinezhad [37]	Two-Stage DEA	Heart Hospital (Iran)	Crisp
2018	Pirani et al. [38]	BCC	Public Hospital (Iran)	Crisp
2018	Serván-Mori et al. [39]	BCC	Maternal Health Service (México)	Crisp
2018	Stefko et al. [40]	CCR	Reginal Health Care (Slovakia)	Crisp
2019	Fuentes et al. [41]	CCR	Public Hospital (Spain)	Crisp
2019	Kocisova et al. [42]	CCR	Reginal Health Care (Slovakia)	Crisp
2019	Serván-Mori et al. [43]	BCC	Maternal Health Service (México)	Crisp
2021	Andrews [44]	BCC	Health Board (New Zealand)	Crisp
2021	Miszczynska and Miszczyński [45]	CCR	Health Care System (Poland)	Crisp
2021	Yüksel [46]	CCR	Health Care System (OECD)	Crisp
2022	Vaňková and Vrabková [47]	CCR	Hospital (Czech and Slovakia)	Crisp
The Current Research		Fuzzy DEA	Hospital (USA)	Uncertain

* CCR: Charnes, Cooper, and Rhodes [25]; BCC: Banker, Charnes, and Cooper [26].

**Table 2:** Description and statistical information of research variables.

Variables		Description	Min	Max
Inputs	TNB	The Number of Beds	49	90
	LRE	Compensation of Medical Doctors, Salaries and Wages of Non-Medical Doctors, Non-Payroll Labor, and Fringe Benefits	3,778,001	9,202,308
	PCSOE	Drugs, Medical Supplies, Food and Food Service Supplies, and Other Supplies and Expenses	2,036,342	4,741,523
Outputs	TNODV	The Number of Patients that Not Require Hospital Admission	35,649	78,483
	TNIDA	The Number of Patients that Require Hospital Admission	3476	7574
	OPSL	The Feedback and Opinion of Patient about the Provided Services	VL	VH

**Table 3:** The linguistic variables and their associated trapezoidal fuzzy number.

Linguistic Variable	Trapezoidal Fuzzy Number
Very Low	(0, 0, 0.1, 0.2)
Low	(0.1, 0.2, 0.2, 0.3)
Medium Low	(0.2, 0.3, 0.4, 0.5)
Medium	(0.4, 0.5, 0.5, 0.6)
Medium High	(0.5, 0.6, 0.7, 0.8)
High	(0.7, 0.8, 0.8, 0.9)
Very High	(0.8, 0.9, 0.9, 1)

**Table 4:** The results of dynamic performance assessment of hospitals (confidence level = 0%).

Hospitals	Windows	Period 1	Period 2	Period 3	Period 4	Period 5	Period 6	Average
Hospital 1	Window 1	0.69954	1.60000	1.28571				1.19509
	Window 2		1.49822	0.95784	1.25000			1.23535
	Window 3			0.95784	1.25000	0.62459		0.94414
	Window 4				1.25000	0.61719	0.87440	0.91387
	Average	0.69954	1.54911	1.06713	1.25000	0.62089	0.87440	1.01018
Hospital 2	Window 1	0.85771	0.62875	0.71094				0.73247
	Window 2		0.76536	0.77909	0.84856			0.79767
	Window 3			0.78860	0.90136	0.81288		0.83428
	Window 4				0.84450	0.81288	0.83861	0.83200
	Average	0.85771	0.69706	0.75954	0.86481	0.81288	0.83861	0.80510
Hospital 3	Window 1	0.90842	0.88028	0.83627				0.87499
	Window 2		0.70965	0.69628	0.87135			0.75909
	Window 3			0.81856	1.05776	0.67136		0.84923
	Window 4				1.05776	0.65933	0.92485	0.88065
	Average	0.90842	0.79497	0.78370	0.99563	0.66534	0.92485	0.84548
Hospital 4	Window 1	1.09588	1.28005	0.55113				0.97569
	Window 2		0.93733	0.66996	0.67735			0.76155
	Window 3			0.70903	0.70578	1.17396		0.86292
	Window 4				0.70578	1.17396	0.89116	0.92363
	Average	1.09588	1.10869	0.64337	0.69630	1.17396	0.89116	0.93489
Hospital 5	Window 1	0.77416	0.62891	0.86885				0.75731
	Window 2		0.70416	0.71245	1.00862			0.80841
	Window 3			0.78428	1.00862	0.70284		0.83191
	Window 4				1.00862	0.70097	0.73921	0.81627
	Average	0.77416	0.66653	0.78852	1.00862	0.70191	0.73921	0.77983
Hospital 6	Window 1	0.70281	1.54583	0.97302				1.07389
	Window 2		1.31824	0.74550	0.68100			0.91491
	Window 3			0.78235	0.76689	0.85136		0.80020
	Window 4				0.76689	0.85136	0.70932	0.77586
	Average	0.70281	1.43204	0.83362	0.73826	0.85136	0.70932	0.87790

**Table 5:** The results of dynamic performance assessment of hospitals (confidence level = 20%).

Hospitals	Windows	Period 1	Period 2	Period 3	Period 4	Period 5	Period 6	Average
Hospital 1	Window 1	0.69760	1.40741	1.15082				1.08527
	Window 2		1.36173	0.88692	1.14286			1.13050
	Window 3			0.88107	1.14286	0.62459		0.88284
	Window 4				1.14286	0.61484	0.84296	0.86689
	Average	0.69760	1.38457	0.97293	1.14286	0.61972	0.84296	0.94344
Hospital 2	Window 1	0.76599	0.58971	0.66467				0.67346
	Window 2		0.76536	0.77909	0.84856			0.79767
	Window 3			0.78860	0.90136	0.80334		0.83110
	Window 4				0.84149	0.80334	0.79600	0.81361
	Average	0.76599	0.67754	0.74412	0.86381	0.80334	0.79600	0.77513
Hospital 3	Window 1	0.84930	0.78614	0.74684				0.79409
	Window 2		0.70845	0.69417	0.79675			0.73313
	Window 3			0.80875	1.02819	0.67136		0.83610
	Window 4				1.02819	0.65676	0.91871	0.86789
	Average	0.84930	0.74730	0.74992	0.95105	0.66406	0.91871	0.81339
Hospital 4	Window 1	0.97566	1.17815	0.51691				0.89024
	Window 2		0.85698	0.66996	0.64963			0.72553
	Window 3			0.70903	0.69538	1.07333		0.82592
	Window 4				0.69538	1.07333	0.87532	0.88135
	Average	0.97566	1.01756	0.63197	0.68013	1.07333	0.87532	0.87566
Hospital 5	Window 1	0.72663	0.58830	0.77593				0.69695
	Window 2		0.70306	0.71038	0.92217			0.77854
	Window 3			0.77532	0.92217	0.70284		0.80011
	Window 4				0.92217	0.69814	0.73120	0.78384
	Average	0.72663	0.64568	0.75388	0.92217	0.70049	0.73120	0.74667
Hospital 6	Window 1	0.70096	1.35997	0.88505				0.98199
	Window 2		1.21642	0.67758	0.67899			0.85767
	Window 3			0.75422	0.75772	0.84120		0.78438
	Window 4				0.75772	0.84120	0.70724	0.76872
	Average	0.70096	1.28820	0.77228	0.73148	0.84120	0.70724	0.84023

**Table 6:** The results of dynamic performance assessment of hospitals (confidence level = 40%).

Hospitals	Windows	Period 1	Period 2	Period 3	Period 4	Period 5	Period 6	Average
Hospital 1	Window 1	0.69760	1.24138	1.02792				0.98897
	Window 2		1.22791	0.82422	1.09454			1.04889
	Window 3			0.83853	1.09454	0.62459		0.85255
	Window 4				1.09303	0.61271	0.82642	0.84405
	Average	0.69760	1.23465	0.89689	1.09404	0.61865	0.82642	0.89471
Hospital 2	Window 1	0.71131	0.58787	0.62037				0.63985
	Window 2		0.76536	0.77909	0.84856			0.79767
	Window 3			0.78860	0.90136	0.79434		0.82810
	Window 4				0.83877	0.79434	0.76787	0.80032
	Average	0.71131	0.67662	0.72935	0.86290	0.79434	0.76787	0.75706
Hospital 3	Window 1	0.79269	0.73003	0.69353				0.73875
	Window 2		0.70845	0.69227	0.76802			0.72291
	Window 3			0.79928	0.99947	0.67136		0.82337
	Window 4				0.99947	0.65444	0.91313	0.85568
	Average	0.79269	0.71924	0.72836	0.92232	0.66290	0.91313	0.78977
Hospital 4	Window 1	0.87205	1.08181	0.51384				0.82257
	Window 2		0.78525	0.66996	0.64775			0.70099
	Window 3			0.70903	0.68752	0.98909		0.79521
	Window 4				0.68752	0.98909	0.86502	0.84721
	Average	0.87205	0.93353	0.63095	0.67427	0.98909	0.86502	0.82748
Hospital 5	Window 1	0.68342	0.58320	0.72055				0.66239
	Window 2		0.70306	0.70852	0.84498			0.75218
	Window 3			0.76719	0.86784	0.70284		0.77929
	Window 4				0.86784	0.69559	0.72565	0.76302
	Average	0.68342	0.64313	0.73209	0.86022	0.69921	0.72565	0.72395
Hospital 6	Window 1	0.69947	1.19964	0.80189				0.90033
	Window 2		1.12348	0.63971	0.67718			0.81346
	Window 3			0.73975	0.74888	0.83139		0.77334
	Window 4				0.74888	0.83139	0.70537	0.76188
	Average	0.69947	1.16156	0.72712	0.72498	0.83139	0.70537	0.80831

**Table 7:** The results of dynamic performance assessment of hospitals (confidence level = 60%).

Hospitals	Windows	Period 1	Period 2	Period 3	Period 4	Period 5	Period 6	Average
Hospital 1	Window 1	0.69760	1.00190	0.80556				0.83502
	Window 2		1.00190	0.71833	1.09243			0.93755
	Window 3			0.82086	1.09243	0.62459		0.84596
	Window 4				1.08775	0.60742	0.79939	0.83152
	Average	0.69760	1.00190	0.78158	1.09087	0.61601	0.79939	0.83122
Hospital 2	Window 1	0.63949	0.58787	0.60378				0.61038
	Window 2		0.76536	0.77909	0.84856			0.79767
	Window 3			0.78860	0.90136	0.77940		0.82312
	Window 4				0.83388	0.77940	0.74863	0.78730
	Average	0.63949	0.67662	0.72382	0.86127	0.77940	0.74863	0.73821
Hospital 3	Window 1	0.77150	0.65632	0.62351				0.68378
	Window 2		0.70845	0.68832	0.76802			0.72159
	Window 3			0.78390	0.97207	0.67136		0.80911
	Window 4				0.96738	0.65255	0.89908	0.83967
	Average	0.77150	0.68239	0.69858	0.90249	0.66195	0.89908	0.76933
Hospital 4	Window 1	0.66585	0.88611	0.51384				0.68860
	Window 2		0.69212	0.66996	0.64347			0.66851
	Window 3			0.70903	0.67378	0.92848		0.77043
	Window 4				0.67378	0.92760	0.84895	0.81678
	Average	0.66585	0.78911	0.63095	0.66367	0.92804	0.84895	0.75443
Hospital 5	Window 1	0.57909	0.58320	0.64780				0.60336
	Window 2		0.70306	0.70590	0.72061			0.70986
	Window 3			0.75944	0.83768	0.70284		0.76665
	Window 4				0.83768	0.68944	0.71322	0.74678
	Average	0.57909	0.64313	0.70438	0.79865	0.69614	0.71322	0.68910
Hospital 6	Window 1	0.69899	0.90918	0.61228				0.74015
	Window 2		0.94415	0.63280	0.67358			0.75017
	Window 3			0.71553	0.73910	0.80667		0.75377
	Window 4				0.73910	0.80667	0.70318	0.74965
	Average	0.69899	0.92666	0.65354	0.71726	0.80667	0.70318	0.75105

**Table 8:** The results of dynamic performance assessment of hospitals (confidence level = 80%).

Hospitals	Windows	Period 1	Period 2	Period 3	Period 4	Period 5	Period 6	Average
Hospital 1	Window 1	0.69760	1.00190	0.73585				0.81178
	Window 2		1.00190	0.71181	1.09102			0.93491
	Window 3			0.80431	1.09102	0.62459		0.83997
	Window 4				1.08277	0.60583	0.78949	0.82603
	Average	0.69760	1.00190	0.75065	1.08827	0.61521	0.78949	0.82385
Hospital 2	Window 1	0.60773	0.58787	0.60321				0.59960
	Window 2		0.76536	0.77909	0.84856			0.79767
	Window 3			0.78860	0.90136	0.77393		0.82130
	Window 4				0.83388	0.77393	0.73845	0.78209
	Average	0.60773	0.67662	0.72363	0.86127	0.77393	0.73845	0.73027
Hospital 3	Window 1	0.77076	0.62373	0.59254				0.66234
	Window 2		0.70845	0.68730	0.76802			0.72126
	Window 3			0.77646	0.96714	0.67136		0.80499
	Window 4				0.95626	0.65255	0.89908	0.83597
	Average	0.77076	0.66609	0.68543	0.89714	0.66195	0.89908	0.76341
Hospital 4	Window 1	0.63418	0.80132	0.51384				0.64978
	Window 2		0.68733	0.66996	0.64347			0.66692
	Window 3			0.70903	0.67118	0.88602		0.75541
	Window 4				0.67001	0.88235	0.83758	0.79665
	Average	0.63418	0.74432	0.63095	0.66155	0.88419	0.83758	0.73213
Hospital 5	Window 1	0.57905	0.58320	0.61563				0.59262
	Window 2		0.70306	0.70590	0.68771			0.69889
	Window 3			0.75505	0.80979	0.70284		0.75590
	Window 4				0.80979	0.68913	0.71020	0.73638
	Average	0.57905	0.64313	0.69219	0.76910	0.69599	0.71020	0.68161
Hospital 6	Window 1	0.69899	0.89414	0.58315				0.72543
	Window 2		0.94415	0.63096	0.67309			0.74940
	Window 3			0.70689	0.73466	0.80667		0.74941
	Window 4				0.73466	0.80667	0.70318	0.74817
	Average	0.69899	0.91914	0.64033	0.71414	0.80667	0.70318	0.74708

**Table 9:** The results of dynamic performance assessment of hospitals (confidence level = 100%).

Hospitals	Windows	Period 1	Period 2	Period 3	Period 4	Period 5	Period 6	Average
Hospital 1	Window 1	0.69760	1.00190	0.70248				0.80066
	Window 2		1.00190	0.70787	1.09102			0.93359
	Window 3			0.78884	1.09102	0.62459		0.83482
	Window 4				1.07789	0.60583	0.78435	0.82269
	Average	0.69760	1.00190	0.73306	1.08664	0.61521	0.78435	0.81979
Hospital 2	Window 1	0.59238	0.58787	0.60321				0.59449
	Window 2		0.76536	0.77909	0.84856			0.79767
	Window 3			0.78860	0.90136	0.76922		0.81973
	Window 4				0.83388	0.76922	0.72866	0.77725
	Average	0.59238	0.67662	0.72363	0.86127	0.76922	0.72866	0.72530
Hospital 3	Window 1	0.77076	0.59200	0.57577				0.64618
	Window 2		0.70845	0.68730	0.76802			0.72126
	Window 3			0.77173	0.96714	0.67136		0.80341
	Window 4				0.95626	0.65255	0.89908	0.83597
	Average	0.77076	0.65022	0.67827	0.89714	0.66195	0.89908	0.75957
Hospital 4	Window 1	0.60370	0.72576	0.51384				0.61443
	Window 2		0.68530	0.66996	0.64347			0.66624
	Window 3			0.70903	0.67118	0.84924		0.74315
	Window 4				0.66676	0.83927	0.82930	0.77844
	Average	0.60370	0.70553	0.63095	0.66047	0.84425	0.82930	0.71237
Hospital 5	Window 1	0.57905	0.58320	0.58431				0.58219
	Window 2		0.70306	0.70590	0.68497			0.69798
	Window 3			0.75294	0.79896	0.70284		0.75158
	Window 4				0.79896	0.68913	0.71020	0.73276
	Average	0.57905	0.64313	0.68105	0.76096	0.69599	0.71020	0.67840
Hospital 6	Window 1	0.69899	0.89366	0.55470				0.71578
	Window 2		0.94415	0.62916	0.67309			0.74880
	Window 3			0.70230	0.73019	0.80667		0.74639
	Window 4				0.73019	0.80667	0.70318	0.74668
	Average	0.69899	0.91890	0.62872	0.71116	0.80667	0.70318	0.74460

## Data Availability

The data used in the study is available with the authors and can be shared upon reasonable requests.
